# Toward the Observation of Dimagnesocene

**DOI:** 10.1021/acsomega.5c06866

**Published:** 2025-12-10

**Authors:** Connor G. Briggs, Stephen M. Goodlett, Henry F. Schaefer

**Affiliations:** † Department of Chemistry and Center for Computational Quantum Chemistry, 138572 University of Georgia, Athens, Georgia 30602, United States; ‡ Institute of Organic Chemistry, Justus Liebig Universität, 35392 Giessen, Germany

## Abstract

We have examined the electronic structure of C_5_H_5_MgMgC_5_H_5_, or dimagnesocene, using high-level coupled-cluster techniques. This research is suitable in light of the remarkable synthesis of the valence-isoelectronic diberyllocene by Boronski, Crumpton, Wales, and Aldridge. The Mg–Mg bond distance is predicted to be 2.758 Å, and the Mg–Mg bond dissociation energy is predicted to be 51.8 kcal/mol. Unique aspects to the present research is our characterization of the ionization energy and the electron affinity of this molecule, the energy of dissociation into two neutral cyclopentadienyl magnesium radicals, the determination of the neutral structure at the CCSD­(T)/cc-pVTZ level, and the computation of the Raman intensities at the MP2/cc-pVDZ level. Apart from mass spectroscopy, the simplest means of experimental detection is gas-phase or matrix-isolated infrared spectroscopy, in which the *A*_2_″ peak at 801 cm^–1^ should be the most prominent, with an intensity of 581 km/mol.

## Graphical abstract

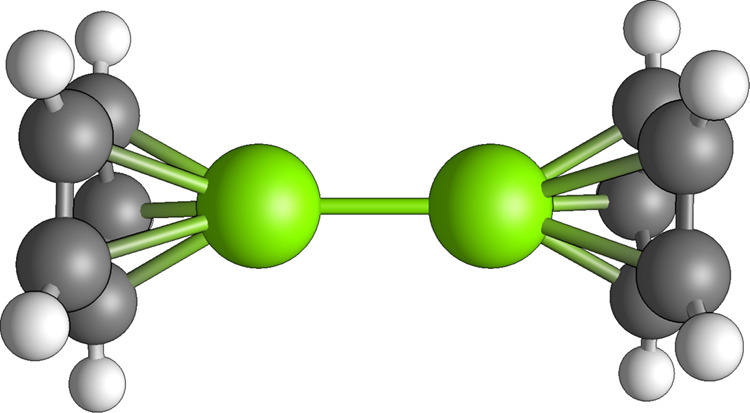

## 1 Introduction

Magnesium­(I) dimers have seen much use as reducing agents since their discovery in 2007.^1,2^ The interest in these compounds is due to how selective they are in what they reduce, targeting mainly unsaturated bonds in organic compounds.^2^ They can also reduce certain metal ions which may be resistant to other more common reagents, such as in the synthesis of diberyllocene,^3^ which used [{(^Mes^Nacnac)­Mg}_2_] to reduce the beryllium ions into the beryllium­(I) state. Most of these dimagnesium reagents consist of a polydentate ligand attached to each of the magnesium centers, such as in [{(^Dip^Nacnac)­Mg}_2_].^1^

There has, of course, been much interest in conventional metallocenes since they were discovered,^4,5^ as they have great potential as catalysts.^6^ This has led many groups to investigate the properties of less common metallocenes. Recently, several groups have synthesized a variety of dimetallocenes, where there are two metal centers between two cyclopentadienyl rings. Examples include a substituted dizincocene,^7^ a lithium–aluminum dimetallocene,^8^ and most spectacularly diberyllocene.^3^ The latter of these is the inspiration for the present research, as dimagnesocene is the next heaviest homometallic group-2 dimetallocene, having the formula (C_5_H_5_Mg)_2_, where C_5_H_5_^–^ is the cyclopentadienyl anion, abbreviated as Cp in this paper.

Previous computational work has been done for this molecule by Xie, Jemmis, and Schaefer.^9^ This was performed using density functional theory methods with double-ζ basis sets. The conclusion drawn by this previous work was that dimagnesocene and its isoelectronic relatives, diberyllocene and dicalcocene, should be stable if synthesized.

## 2 Theoretical Methods

The goal of this study is to confirm our understanding of the properties of dimagnesocene. Previous calculations performed in 2005 used density functional theory methods,^9^ which take approximations to a variety of effects that are handled more rigorously in coupled-cluster methods, which explicitly include electron correlation. Energies for dimagnesocene were initially computed using MolPro,^10–12^ including geometries and harmonic frequencies at the CCSD^13–15^ /cc-pVDZ^16,17^ level. The higher-level structures for CCSD­(T)^13–15,18^ /cc-pVDZ and CCSD/cc-pVTZ^16,17^ were computed using OptAVC, a wrapper around Psi4’s OptKing^19^ module, allowing the use of MolPro to compute energies for finite differences with multiple jobs on a supercomputer. The structure for CCSD­(T)/cc-pVTZ was computed using MolPro as well, but with symmetry restricted to the *C*_2v_ point group. The ionization energy, electron affinity, and geometries of the C_5_H_5_Mg radical were computed using MolPro’s RHF-UCCSD^20–23^ /cc-pVDZ, RHF-UCCSD/cc-pVTZ, RHF-UCCSD­(T)/cc-pVDZ, and RHF-UCCSD­(T)/cc-pVTZ methods. Raman intensities were computed at the MP2/cc-pVDZ level using MolPro’s potential energy surface generator^24–27^ and vibrational self-consistent field and vibrational configuration interaction methods.^28–33^

A few energy values were not computed in the previous work. The current work also extends some of the DFT calculations from the previous work for comparison. These were done with the same functionals and basis sets as in the work by Xie, Jemmis, and Schaefer.^9^ This was done in MolPro with the B3LYP and BP86 functionals^34^ using a mixed basis set consisting of the McLean–Chandler double-ζ (12s9p/6s5p)^35^ basis with a set of d-type polarization functions with exponent α = 0.175 for magnesium, and the Huzinaga–Dunning double-ζ basis^36,37^ with a set of d-type polarization functions on carbon with exponent α_d_ = 0.75 and a set of p-type polarization functions on hydrogen with exponent α_p_ = 0.75. The particular values computed were the ionization energies, electron affinities, and various bond dissociation energies.

While magnesium is a rather light element, it lies close to the threshold where relativistic effects can become important. As a result, the geometry was also computed with CCSD/cc-pVDZ with the exact 2-component (X2C) correction,^38–41^ though the difference from the uncorrected CCSD/cc-pVDZ Mg–Mg bond was only 0.004 Å, and was even lower for the other geometric parameters. The difference in bond dissociation energy was similarly small, less than 0.1 kcal/mol. As a result, relativistic corrections were not considered in this work.

## 3 Results and Discussion

The qualitative structure for dimagnesocene is shown in Figure 1. Some geometric parameters are listed in Table 1. The definition of the Mg-ring distance is the distance between a magnesium atom and the plane of its closest cyclopentadienyl ring. The definition of the *∠*H-ring plane is the angle between any C–H bond and the plane of the cyclopentadienyl ring, with positive angles moving toward the center of the molecule. A graphic of this and the other geometric parameters is given in Figure 2. As the values in Table 1 indicate, there is a serendipitous agreement between the present high-level coupled-cluster predictions and the earlier DFT results. This could not have been anticipated.

**Figure 1:**
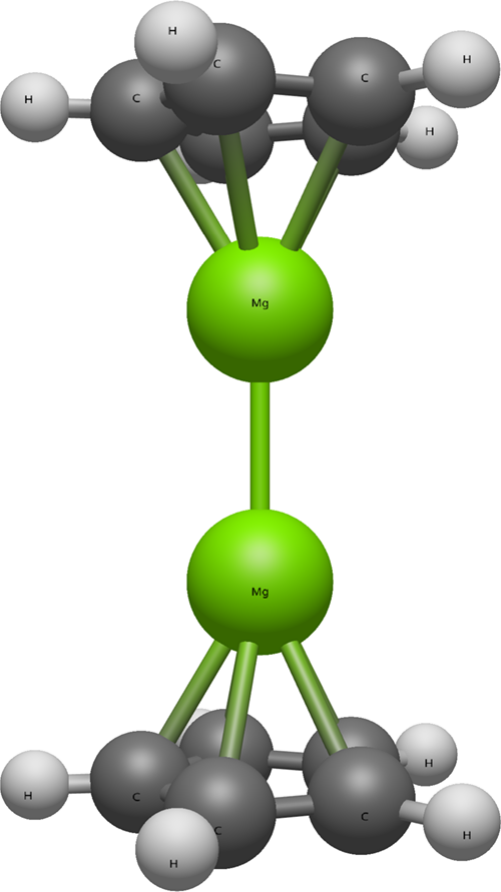
Molecular structure of dimagnesocene. Different levels of theory predict slightly different bond distances but all predict the same qualitative structure. Here and in the rest of the paper, gray atoms represent carbon, green atoms represent magnesium, and white atoms represent hydrogen.

**Table 1:** Comparison of the Geometric Parameters of Dimagnesocene^a^

	CCSD/DZ	CCSD/TZ	CCSD(T)/DZ	CCSD(T)/TZ	B3LYP^9^	BP86 ^9^
Mg–Mg (Å)	2.781	2.765	2.776	2.758	2.766	2.786
Mg-ring center (Å)	2.051	1.997	2.050	2.044	2.042	2.041
Mg–C (Å)	2.385	2.333	2.386	2.334	2.376	2.378
C–C (Å)	1.418	1.413	1.435	1.418	1.428	1.436
*∠*H-ring plane	0.89°	0.44°	0.53°	0.43°	1.3°	1.2°

a The values of the *∠*H-ring plane refer to the angle between the plane of the rings and the hydrogen atoms surrounding them, shown graphically in Figure 2. The values for Mg-ring center are the distances from a magnesium atom to the center of the nearest cyclopentadienyl ring. The Mg–C values are the distances between a magnesium atom and one carbon atom of the nearest ring.

**Figure 2:**
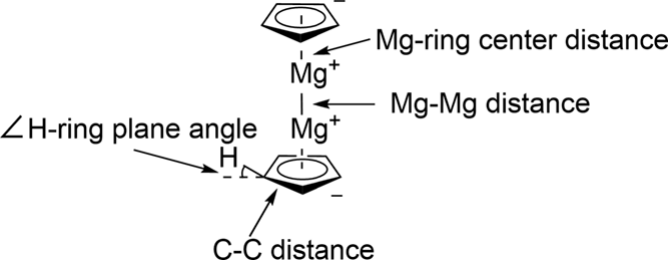
Graphical explanation of the geometric parameters. Positive angles for the *∠*H-ring plane parameter indicate the hydrogens being closer to the center of mass of the molecule, while negative angles indicate the hydrogens being further away.

The harmonic vibrational frequencies for dimagnesocene at the CCSD/cc-pVDZ level are reported in Table 2. The point group used for the assignment of the modes is *D*_5h_, though the values were calculated in *C*_2v_ and then correlated to the appropriate irreducible representations by both looking at the symmetry species in *C*_2v_ provided by MolPro and separately visualizing the modes. The infrared intensities are reported only for the IR-active modes, namely, those of either *A*_2_″ or *E*_1_′ symmetry. The lowest energy vibration, the ν_6_ mode, corresponds to conrotatory torsion of the two magnesocene structures around the Mg–Mg bond, indicating that there is a very low energy barrier to this motion. The frequencies calculated in previous work by Xie, Schaefer, and Jemmis^9^ at the B3LYP level are also included for comparison. The table shows that there is a red-shift in the DFT values from what is calculated using coupled-cluster methods. The only DFT frequency that is not red-shifted is the ν_6_ mode, which is likely due to how sensitive this mode is to slight differences in geometry.

**Table 2:** Harmonic Vibrational Frequencies Are Given in cm^–1^ ^a^

		CCSD/cc-pVDZ		B3LYP^9^	
*a*_1_ *′*	ν_1_	132		128	
	ν_2_	466		453	
	ν_3_	807		788	
	ν_4_	1153		1135	
	ν_5_	3278		3248	
*a*_1_″	ν_6_	3		21	
	ν_7_	1283		1269	
*a*_2_′	ν_8_	1283		1269	
*a*_2_″	ν_9_	370	(148)	364	(154)
	ν_10_	801	(581)	781	(630)
	ν_11_	1152	(28)	1134	(21)
	ν_12_	3278	(3)	3248	(2)
*e*_1_′	ν_13_	39	(0)	38	(2)
	ν_14_	254	(2)	242	(2)
	ν_15_	778	(0)	761	(1)
	ν_16_	1031	(29)	1019	(40)
	ν_17_	1482	(2)	1457	(2)
	ν_18_	3264	(6)	3236	(7)
*e*_1_″	ν_19_	100		97	
	ν_20_	250		237	
	ν_21_	779		763	
	ν_22_	1031		1019	
	ν_23_	1483		1457	
	ν_24_	3264		3236	
*e*_2_′	ν_25_	626		620	
	ν_26_	860		855	
	ν_27_	885		871	
	ν_28_	1078		1068	
	ν_29_	1418		1386	
	ν_30_	3246		3220	
*e*_2_″	ν_31_	626		620	
	ν_32_	860		855	
	ν_33_	884		870	
	ν_34_	1078		1068	
	ν_35_	1418		1386	
	ν_36_	3245		3220	

a Infrared intensities in km/mol are given in parentheses where they are nonzero. The intensities for the ν_13_ and ν_15_ modes are greater than zero but round down to zero.

By far, the most prominent peak should lie around 801 cm^–1^ with an intensity of 581 km/mol, neglecting anharmonicity. The second most prominent peak should lie around 370 cm^–1^ with an intensity of 148 km/mol. Graphic representations of these two modes can be found in Figures 3 and 4, and the rest can be found in the Supporting Information. These frequencies can be scaled by 0.9473 to account for anharmonicity, as suggested by the National Institute of Standards and Technology for CCSD/cc-pVDZ calculations.^42^ This would mean that the most prominent peak should lie at 759 cm^–1^ and the second most prominent peak should lie at 351 cm^–1^ after accounting for anharmonicity.

**Figure 3:**
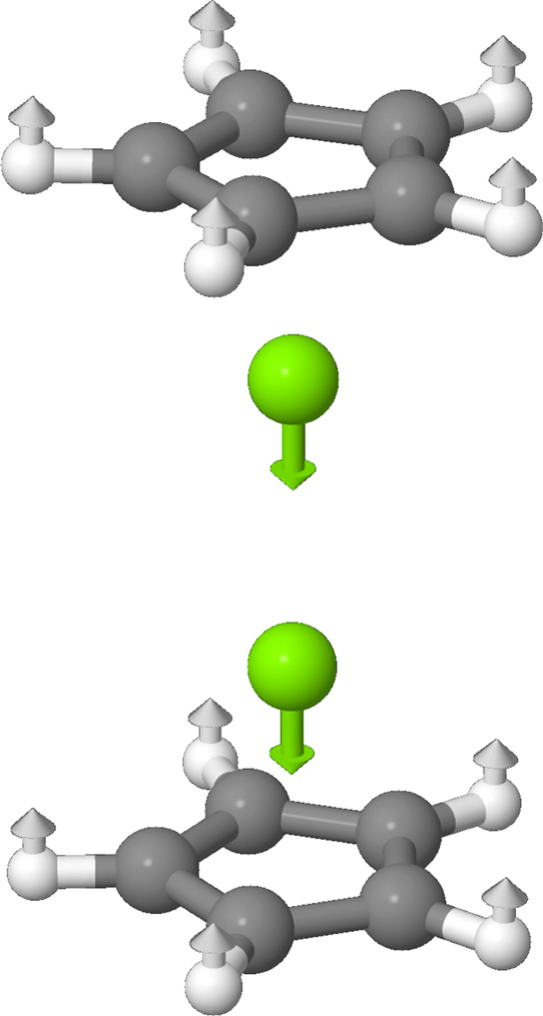
Graphic representation of the ν_9_ (*a*_2_″) mode at about 370 cm^–1^.

**Figure 4:**
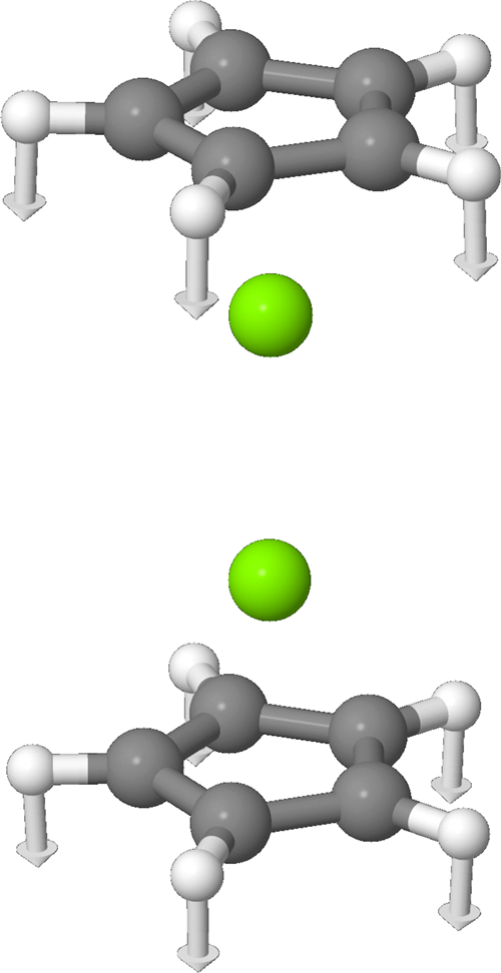
Graphic representation of the ν_10_ (*a*_2_″) mode at about 801 cm^–1^.

Raman intensities were also computed at the MP2/cc-pVDZ level by using an exciting frequency of 680 nm and a polarization angle of 90°. These are reported in Table 3 along with the corresponding CCSD frequencies. Only the Raman-active modes are reported. As shown in the table, there should be three relatively strong Raman signals corresponding to ν_4_, ν_5_, and ν_30_ at 1153, 3278, and 3246 cm^–1^, respectively, based on the CCSD/cc-pVDZ harmonic frequencies. Schematic representations of these modes can be found in the Supporting Information.

**Table 3:** Raman Intensities in Å^4^/amu and Depolarization Ratios Were Calculated at the MP2/cc-pVDZ Level^a^

		CCSD freq	MP2 freq	MP2 Raman intens.	depol.
*a*_1_′	ν_1_	132	130	(26)	0.15
	ν_2_	466	469	(6)	0.14
	ν_3_	807	811	(1)	0.46
	ν_4_	1153	1140	(103)	0.00
	ν_5_	3278	3276	(454)	0.12
*e*_1_″	ν_19_	100	100	(9)	0.75
	ν_20_	250	254	(16)	0.75
	ν_21_	779	782	(5)	0.75
	ν_22_	1031	1027	(0)	0.75
	ν_23_	1483	1460	(0)	0.75
	ν_24_	3264	3294	(37)	0.75
*e*_2_′	ν_25_	626	624	(1)	0.75
	ν_26_	860	848	(1)	0.75
	ν_27_	885	882	(0)	0.75
	ν_28_	1078	1081	(14)	0.75
	ν_29_	1418	1433	(7)	0.75
	ν_30_	3246	3277	(155)	0.75

a The MP2 anharmonic frequencies are shown next to the corresponding CCSD/cc-pVDZ harmonic frequencies for reference.

The computed ionization energies and electron affinities are listed in Table 4. These are computed adiabatically by adding or removing an electron from the neutral geometry found with the same method and basis set. The strength estimates of Xie, Schaefer, and Jemmis^9^ were also replicated for comparison, where the energy change of the following reaction is used to estimate this property:

$${\left(C_{5}H_{5}\mathrm{Mg}\right)}_{2}+H_{2}\to 2C_{5}H_{5}\mathrm{MgH}$$ 1

$$\Delta E_{\mathrm{Mg}-\mathrm{Mg}}=2E_{C_{5}H_{5}\mathrm{MgH}}-E_{H_{2}}-E_{(C_{5}H_{5}\mathrm{Mg}{)}_{2}}$$ 2

**Table 4:** Bond Dissociation Energies, Electron Affinities, Ionization Energies, and Reaction Energies for Dimagnesocene

	CCSD/DZ	CCSD/TZ	CCSD(T)/DZ	CCSD(T)/TZ	B3LYP	BP86
ionization energy (eV)	7.34	7.37	7.37	7.42	7.49	7.49
electron affinity (eV)	0	0	0	0	0	0
hydrogen addition energy (kcal/mol)	14.6	11.4	16.0	13.0	10.0 ^9^	12.4 ^9^
Mg–Mg bond dissociation energy (kcal/mol)	48.8	50.5	50.0	51.8	47.3	45.3
H–Mg bond dissociation energy (kcal/mol)	68.9	73.7	68.8	73.6	73.2	71.7

As this is a reaction energy and not a true bond strength, it may be biased by the H–H and H–Mg bond energies. This estimate can then be compared to the bond dissociation energy computed in a more conventional way, expressed in the following equation.

$${\left(C_{5}H_{5}\mathrm{Mg}\right)}_{2}\to 2C_{5}H_{5}\mathrm{Mg}$$ 3

Similarly, we can bridge the gap between the previous work’s estimates for the bond energy and the current work’s estimate by considering the energy of the following equation, which will be reported as the H–Mg bond dissociation energy.

$$C_{5}H_{5}\mathrm{MgH}\to C_{5}H_{5}\mathrm{Mg}+H$$ 4

The structures of the C_5_H_5_MgH intermediates are given in the Supporting Information, and an example of the structure can be seen in Figure 5. The structures and energies for this intermediate were predicted at the same level as those of the corresponding dimagnesocene structure. The structure and energies for the hydrogen molecule were computed with the same basis set as the corresponding dimagnesocene but were only treated with CCSD due to having only two electrons, giving a rigorously zero triples contribution.

**Figure 5:**
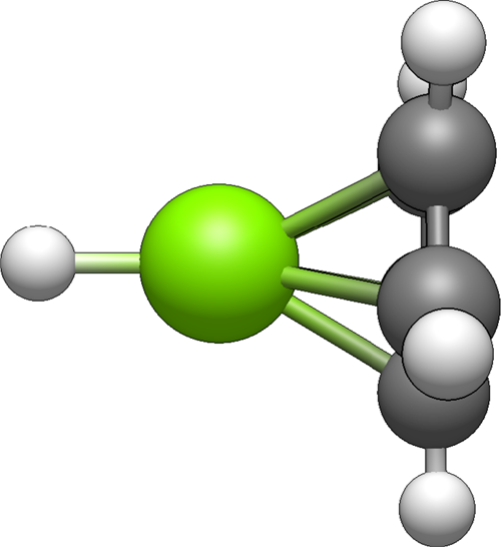
Qualitative structure of the cyclopentadienyl magnesium hydride used for the bond strength computations.

From these results, the addition of hydrogen to this molecule should be expected to be endothermic. This agrees with previous DFT calculations,^9^ though the inclusion of electron correlation seems to increase the endothermicity further than previously predicted. The Mg–Mg bond dissociation energy is expected to be relatively high, indicating that it should be somewhat stable. The large H–Mg bond dissociation energy seems to indicate that the formation of the cyclopentadienyl magnesium hydride is a major contributor to the total hydrogen addition energy, which means that this method of estimating the bond strength may not be the most sound. Similarly, the high ionization energy also points to this being relatively stable. The computed electron affinities are all negative, which indicates that adding an electron is energetically unfavorable. Thus, the true electron affinities are all zero. Altogether, this shows that coupled-cluster methods predict that dimagnesocene will be viable, somewhat more so than DFT predicts.

## Conclusions

This research indicates that, if synthesized, dimagnesocene should be stable, and its infrared spectrum should show two easily characterized peaks corresponding to vibrational transitions with *a*_2_″ symmetry. The impact of including explicit electron correlation through coupled-cluster theory seems to give quite similar results to previous DFT calculations. The only major differences between the previous work and the current work are in the size of *∠*H-ring plane and the Mg–Mg bond dissociation energy predicted by BP86, which is 6.6 kcal/mol lower than the value predicted by CCSD­(T)/cc-pVTZ. Compared to the experimental value of 3.890 Å^43^ for the diatomic magnesium molecule, there is a significant decrease in the Mg–Mg bond distance to 2.806 Å caused by the inclusion of cyclopentadiene rings. This bond distance is slightly shorter than what is seen in other dimeric magnesium compounds,^44^ which start at about 2.808 Å for [{(^Mes^Nacnac)­Mg}_2_], but can get up to 3.196 Å for [{(^Dip^Nacnac)­Mg­(DMAP)}_2_]. This may be due to the difference in how the particular ligands interact with the magnesium centers, as the substituted NacNac ligands form a bond through the imine groups, while in dimagnesocene, the interaction is only with an aromatic ring. Despite this difference in bond length, the calculated bond dissociation energies for dimagnesocene agree well with calculations on these known compounds, which lie around 45 kcal/mol,^45^ lending more credence to the potential stability of dimagnesocene. Similarly, the predicted ionization energy of dimagnesocene lies very close to the appearance energy of magnesocene, which has been experimentally determined to be 7.76 ± 0.1 eV.^46^

## Supplementary Material



## Notes

Structures in XYZ format for dimagnesocene, cyclopentadienyl magnesium­(I), cyclopentadienyl magnesium hydride, and hydrogen presented in this study at different levels of theory, structures containing a comment line indicating the level of theory and the optimized energy of the structure, and graphical representations of the normal modes, each labeled with their frequency and symmetry (PDF)
