# Supplementary material for: Toward the Observation of Dimagnesocene
Source: ACS Omega. 2025 Dec 10;10(50):61508–13. doi: 10.1021/acsomega.5c06866 (PMC12750217; doi:10.1021/acsomega.5c06866)
Supplement: Supplementary file 1 [file ao5c06866_si_001.pdf]

Toward the Observation of Dimagnesocene

Connor G. Briggs,[*,†] Stephen M. Goodlett,[‡] and Henry F. Schaefer III[†]

†*Department of Chemistry and Center for Computational Quantum Chemistry, University of Georgia, Athens, GA, USA 30602*

‡*Institute of Organic Chemistry, Justus Liebig Universität, 35392 Giessen, Germany*

E-mail: Connor.Briggs@uga.edu

Molecular Geometries

All coordinates are in Angstrom. All energies are in Hartrees.

Dimagnesocene Geometries

```
  22
CCSD/CC-PVDZ Dimagnesocene ENERGY=-785.27864450
C          0.9844277735      -0.7152286432      -3.4413849797
C         -0.3760179500      -1.1572642545      -3.4413849797
C         -1.2168196470      -0.0000000000      -3.4413849797
C         -0.3760179500       1.1572642545      -3.4413849797
C          0.9844277735       0.7152286432      -3.4413849797
C          0.9844277735      -0.7152286432       3.4413849797
C         -0.3760179500      -1.1572642545       3.4413849797
C         -1.2168196470      -0.0000000000       3.4413849797
C         -0.3760179500       1.1572642545       3.4413849797
C          0.9844277735       0.7152286432       3.4413849797
H          1.8677033494      -1.3569659130      -3.4713606471
H         -0.7133991986      -2.1956169688      -3.4713606471
H         -2.3086083016      -0.0000000000      -3.4713606471
H         -0.7133991986       2.1956169688      -3.4713606471
H          1.8677033494       1.3569659130      -3.4713606471
H          1.8677033494      -1.3569659130       3.4713606471
H         -0.7133991986      -2.1956169688       3.4713606471
H         -2.3086083016      -0.0000000000       3.4713606471
H         -0.7133991986       2.1956169688       3.4713606471
H          1.8677033494       1.3569659130       3.4713606471
```

Mg	0.0000000000	-0.0000000000	-1.3905484112
Mg	0.0000000000	-0.0000000000	1.3905484112

22

CCSD/CC-PVTZ Dimagnesocene ENERGY=-785.83205632

C	0.9721017323	-0.7062732501	-3.3821380367
C	-0.3713098212	-1.1427741239	-3.3821380367
C	-1.2015838221	0.0000000000	-3.3821380367
C	-0.3713098212	1.1427741239	-3.3821380367
C	0.9721017323	0.7062732501	-3.3821380367
C	0.9721017323	-0.7062732501	3.3821380367
C	-0.3713098212	-1.1427741239	3.3821380367
C	-1.2015838221	0.0000000000	3.3821380367
C	-0.3713098212	1.1427741239	3.3821380367
C	0.9721017323	0.7062732501	3.3821380367
H	1.8404023049	-1.3371305431	-3.4072458594
H	-0.7029711275	-2.1635226662	-3.4072458594
H	-2.2748623548	0.0000000000	-3.4072458594
H	-0.7029711275	2.1635226662	-3.4072458594
H	1.8404023049	1.3371305431	-3.4072458594
H	1.8404023049	-1.3371305431	3.4072458594
H	-0.7029711275	-2.1635226662	3.4072458594
H	-2.2748623548	0.0000000000	3.4072458594
H	-0.7029711275	2.1635226662	3.4072458594
H	1.8404023049	1.3371305431	3.4072458594
Mg	-0.0000000000	0.0000000000	-1.3823746129
Mg	-0.0000000000	0.0000000000	1.3823746129

22

CCSD(T)/CC-PVDZ Dimagnesocene ENERGY=-785.34632692

C	0.9877742311	-0.7176599870	-3.4381840367
C	-0.3772961831	-1.1611982513	-3.4381840367
C	-1.2209560961	0.0000000000	-3.4381840367
C	-0.3772961831	1.1611982513	-3.4381840367
C	0.9877742311	0.7176599870	-3.4381840367
C	0.9877742311	-0.7176599870	3.4381840367
C	-0.3772961831	-1.1611982513	3.4381840367
C	-1.2209560961	0.0000000000	3.4381840367
C	-0.3772961831	1.1611982513	3.4381840367
C	0.9877742311	0.7176599870	3.4381840367
H	1.8725823465	-1.3605107120	-3.4687232717
H	-0.7152628096	-2.2013525740	-3.4687232717
H	-2.3146390738	0.0000000000	-3.4687232717
H	-0.7152628096	2.2013525740	-3.4687232717
H	1.8725823465	1.3605107120	-3.4687232717
H	1.8725823465	-1.3605107120	3.4687232717
H	-0.7152628096	-2.2013525740	3.4687232717
H	-2.3146390738	0.0000000000	3.4687232717
H	-0.7152628096	2.2013525740	3.4687232717
H	1.8725823465	1.3605107120	3.4687232717
Mg	0.0000000000	0.0000000000	-1.3882299487
Mg	0.0000000000	0.0000000000	1.3882299487

22

CCSD(T)/CC-PVTZ ENERGY=-785.92893235

C	0.9756620517	-0.7088599735	-3.3767520027
C	-0.3726697422	-1.1469595304	-3.3767520027

C	-1.2059846189	0.0000000000	-3.3767520027
C	-0.3726697422	1.1469595304	-3.3767520027
C	0.9756620517	0.7088599735	-3.3767520027
C	0.9756620517	-0.7088599735	3.3767520027
C	-0.3726697422	-1.1469595304	3.3767520027
C	-1.2059846189	0.0000000000	3.3767520027
C	-0.3726697422	1.1469595304	3.3767520027
C	0.9756620517	0.7088599735	3.3767520027
H	1.8456293624	-1.3409282227	-3.4014420779
H	-0.7049676858	-2.1696674408	-3.4014420779
H	-2.2813233531	0.0000000000	-3.4014420779
H	-0.7049676858	2.1696674408	-3.4014420779
H	1.8456293624	1.3409282227	-3.4014420779
H	1.8456293624	-1.3409282227	3.4014420779
H	-0.7049676858	-2.1696674408	3.4014420779
H	-2.2813233531	0.0000000000	3.4014420779
H	-0.7049676858	2.1696674408	3.4014420779
H	1.8456293624	1.3409282227	3.4014420779
Mg	0.0000000000	0.0000000000	-1.3789896461
Mg	0.0000000000	0.0000000000	1.3789896461

Geometries for Bond Dissociation Energy

11

UCCSD/CC-PVDZ Magnesium Cyclopentadienyl ENERGY=-392.60041604

C	0.9844242961	-0.7152261167	-0.5577273747
C	-0.3760166217	-1.1572601665	-0.5577273747
C	-1.2168153486	0.0000000000	-0.5577273747

C	-0.3760166217	1.1572601665	-0.5577273747
C	0.9844242961	0.7152261167	-0.5577273747
H	1.8676072419	-1.3568960868	-0.5886918548
H	-0.7133624888	-2.1955039877	-0.5886918548
H	-2.3084895062	0.0000000000	-0.5886918548
H	-0.7133624888	2.1955039877	-0.5886918548
H	1.8676072419	1.3568960868	-0.5886918548
Mg	-0.0000000000	0.0000000000	1.5001500854

11

UCCSD/CC-PVTZ Magnesium Cyclopentadienyl ENERGY=-392.87583679

C	0.9722451373	-0.7063775079	-0.5431569280
C	-0.3713647533	-1.1429428594	-0.5431565265
C	-1.2017613046	0.0000000000	-0.5431564034
C	-0.3713647533	1.1429428594	-0.5431565265
C	0.9722451373	0.7063775079	-0.5431569280
H	1.8403492851	-1.3370921078	-0.5700480459
H	-0.7029510586	-2.1634605195	-0.5700474311
H	-2.2747970372	0.0000000000	-0.5700471365
H	-0.7029510586	2.1634605195	-0.5700474311
H	1.8403492851	1.3370921078	-0.5700480459
Mg	0.0000002894	0.0000000000	1.4602815201

11

UCCSD(T)/CC-PVDZ Magnesium Cyclopentadienyl ENERGY=-392.63337504

C	0.9877232992	-0.7176229953	-0.5579711005
C	-0.3772767477	-1.1611384006	-0.5579710485
C	-1.2208931636	0.0000000000	-0.5579710449
C	-0.3772767477	1.1611384006	-0.5579710485

C	0.9877232992	0.7176229953	-0.5579711005
H	1.8724234827	-1.3603953275	-0.5895642774
H	-0.7152021449	-2.2011658685	-0.5895640826
H	-2.3144427445	0.0000000000	-0.5895642454
H	-0.7152021449	2.2011658685	-0.5895640826
H	1.8724234827	1.3603953275	-0.5895642774
Mg	0.0000000328	0.0000000000	1.5009331079

11

RCCSD(T)/CC-PVTZ Magnesium Cyclopentadienyl ENERGY=-392.92316219

C	0.9757517805	-0.7089251175	-0.5432051329
C	-0.3727039155	-1.1470649140	-0.5432053916
C	-1.2060953860	0.0000000000	-0.5432054760
C	-0.3727039155	1.1470649140	-0.5432053916
C	0.9757517805	0.7089251175	-0.5432051329
H	1.8455114571	-1.3408425124	-0.5696658343
H	-0.7049225337	-2.1695287295	-0.5696661847
H	-2.2811774791	0.0000000000	-0.5696663760
H	-0.7049225337	2.1695287295	-0.5696661847
H	1.8455114571	1.3408425124	-0.5696658343
Mg	-0.0000001852	0.0000000000	1.4603225982

Geometries for Hydrogen Addition Reaction

12

CCSD/CC-PVDZ Magnesium Cyclopentadienyl Hydride ENERGY=-393.20948996

C	-1.2177574546	0.0000000000	-0.5821473605
C	-0.3763228107	1.1581364417	-0.5821792235
C	0.9851478839	0.7157674897	-0.5822463982

C	0.9851478839	-0.7157674897	-0.5822463982
C	-0.3763228107	-1.1581364417	-0.5821792235
H	-2.3094444696	0.0000000000	-0.6134569215
H	-0.7136735478	2.1963902352	-0.6135432562
H	1.8683380945	1.3574422208	-0.6136955549
H	1.8683380945	-1.3574422208	-0.6136955549
H	-0.7136735478	-2.1963902352	-0.6135432562
Mg	0.0000580292	0.0000000000	1.4357735517
H	-0.0000051889	0.0000000000	3.1349306501

12

CCSD/CC-PVTZ Magnesium Cyclopentadienyl Hydride ENERGY=-393.49307837

C	3.3376824481	0.9664303865	-0.7148571333
C	3.3388834035	0.9664935575	0.6988872986
C	3.3385897331	-0.3780381682	1.1358189852
C	3.3372072429	-1.2090676107	-0.0078868942
C	3.3366465219	-0.3781402628	-1.1516675736
H	3.3654673922	1.8345609095	-1.3456841300
H	3.3677415149	1.8346803568	1.3295886592
H	3.3671866083	-0.7096188074	2.1564079418
H	3.3645695401	-2.2822016501	-0.0078622054
H	3.3635070486	-0.7098120531	-2.1722740785
Mg	1.3688932980	-0.0054932642	-0.0062651032
H	-0.3246371072	-0.0046553203	-0.0048269948

12

CCSD(T)/CC-PVDZ Magnesium Cyclopentadienyl Hydride ENERGY=-393.24223302

C	0.4645652701	0.9882136321	-0.7186111312
C	0.4657830614	0.9882778376	0.7177292674

```
C          0.4654903072      -0.3777438257       1.1616450769
C          0.4640915884      -1.2220564051      -0.0003413612
C          0.4635198844      -0.3778475383      -1.1624037380
H          0.4963534208       1.8728712959      -1.3614516047
H          0.4986592196       1.8729929687       1.3604359449
H          0.4981092295      -0.7156392790       2.2016625782
H          0.4954634793      -2.3156237717      -0.0003191506
H          0.4943783427      -0.7158358202      -2.2024447751
Mg        -1.5532112187       0.0007448433       0.0013222890
H         -3.2532025848       0.0016460623       0.0027766044

  12
CCSD(T)/CC-PVTZ  ENERGY=-393.54030664
C          0.9764446502      -0.7094285748      -0.5683878723
C         -0.3729686734      -1.1478795529      -0.5683878776
C         -1.2069519578       0.0000000000      -0.5683878674
C         -0.3729686734       1.1478795529      -0.5683878776
C          0.9764446502       0.7094285748      -0.5683878723
H          1.8462693987      -1.3413933084      -0.5957792880
H         -0.7052121413      -2.1704199229      -0.5957793171
H         -2.2821144950       0.0000000000      -0.5957792886
H         -0.7052121413       2.1704199229      -0.5957793171
H          1.8462693987       1.3413933084      -0.5957792880
Mg         0.0000000030       0.0000000000       1.3996686609
H         -0.0000000445       0.0000000000       3.0935729042
```

These hydrogen parameters also represent those for CCSD(T). Since there are only two electrons, the triples contribution is rigorously zero.

```
2
CCSD/CC-PVDZ  ENERGY=-1.16367298
H          0.0000000000        0.0000000000       -0.3804466507
H          0.0000000000        0.0000000000        0.3804466507

2
CCSD/CC-PVTZ  ENERGY=-1.17233669
H          0.0000000000        0.0000000000       -0.3713114394
H          0.0000000000        0.0000000000        0.3713114394
```

Normal Modes

Here is a list of the normal modes and their schematic representations.

Modes with A_1' Symmetry

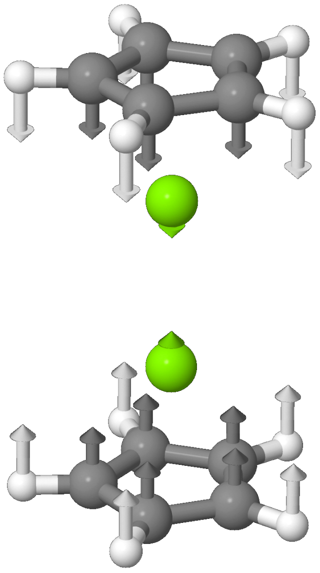

Figure 1: $\nu_1(a_1')$ mode at 132 cm^{-1}

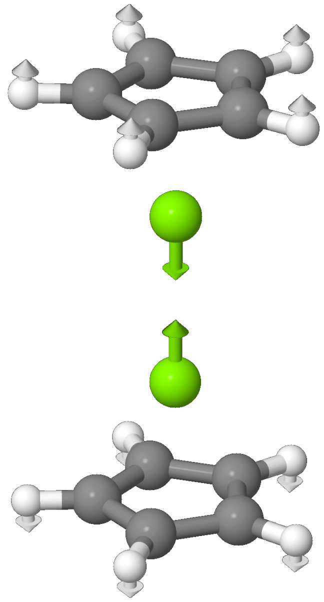

Figure 2: $\nu_2(a_1')$ mode at 466 cm^{-1}

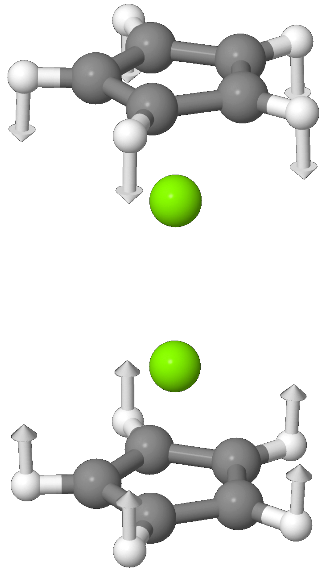

Figure 3: $\nu_3(a_1')$ mode at 807 cm^{-1}

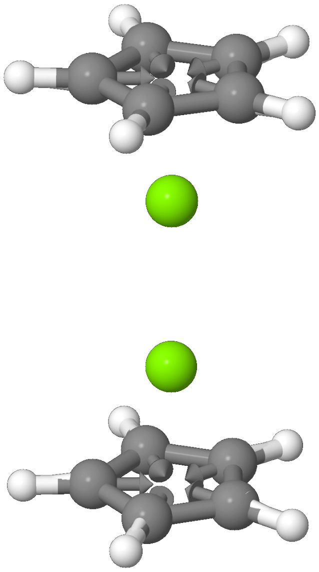

Figure 4: $\nu_4(a_1')$ mode at 1153 cm^{-1}

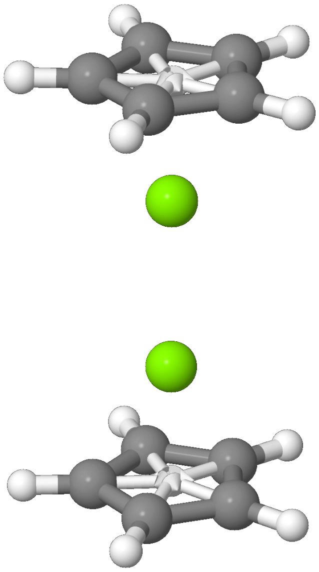

Figure 5: $\nu_5(a_1')$ mode at 3278 cm^{-1}

Modes with A_1'' Symmetry

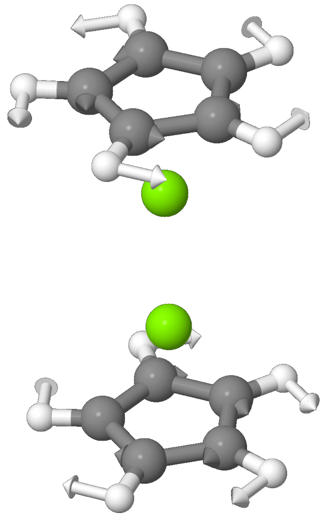

Figure 6: $\nu_6(a_1'')$ mode at 3 cm^{-1}

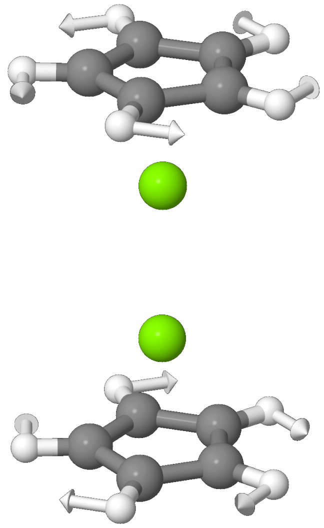

Figure 7: $\nu_7(a_1'')$ mode at 1283 cm^{-1}

Mode with A_2' Symmetry

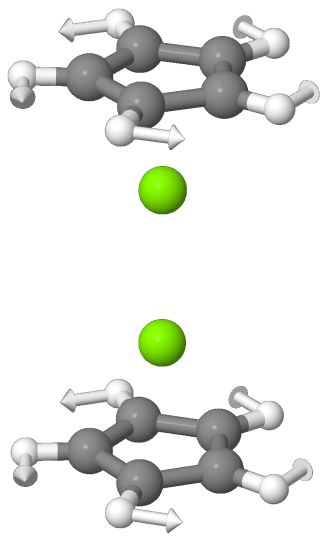

Figure 8: $\nu_8(a_2')$ mode at 1283 cm^{-1}

Modes with A_2'' Symmetry

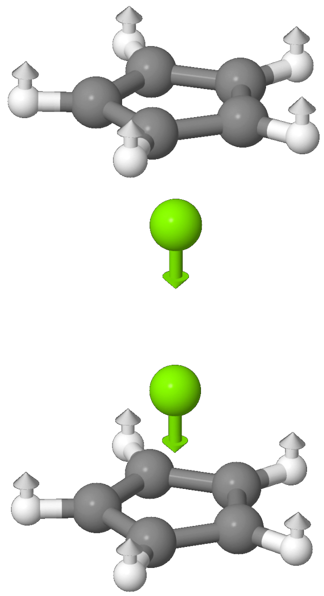

Figure 9: $\nu_9(a_2'')$ mode at 370 cm^{-1}

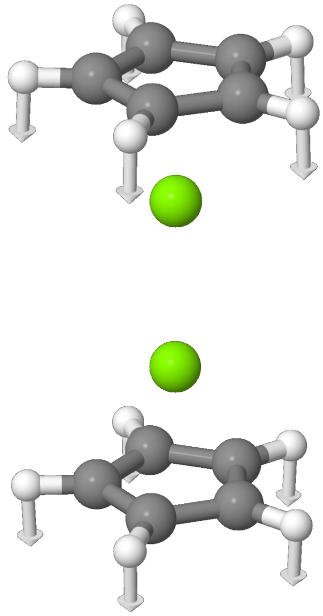

Figure 10: $\nu_{10}(a_2'')$ mode at 801 cm^{-1}

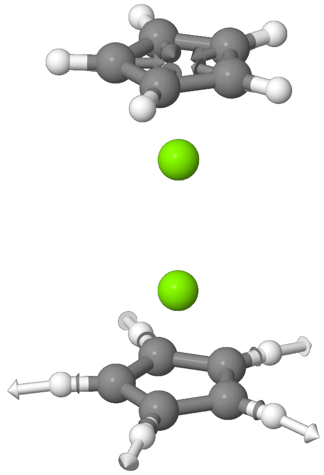

Figure 11: $\nu_{11}(a_2'')$ mode at 1152 cm^{-1}

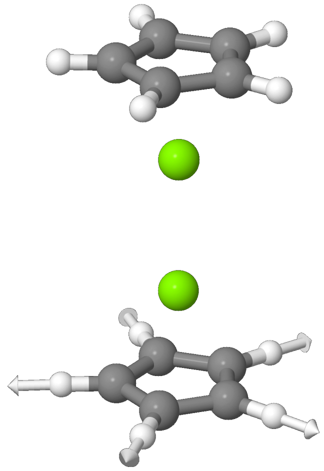

Figure 12: $\nu_{12}(a_2'')$ mode at 3278 cm^{-1}

Modes with E_1' Symmetry

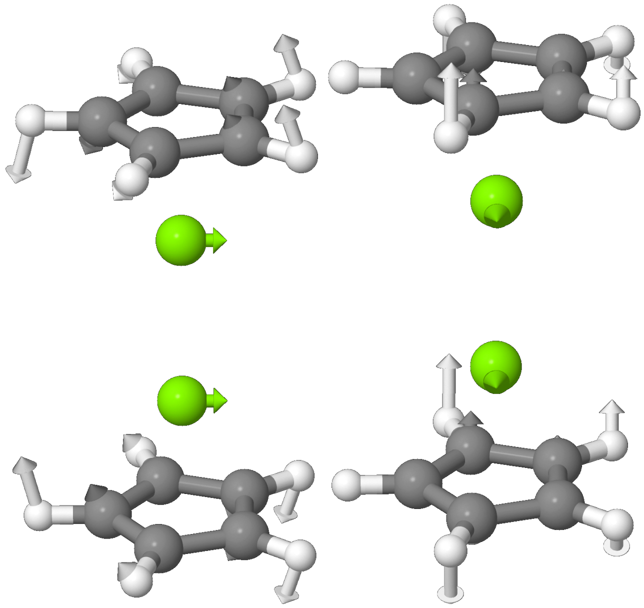

Figure 13: $\nu_{13}(e_1')$ modes at 39 cm^{-1}

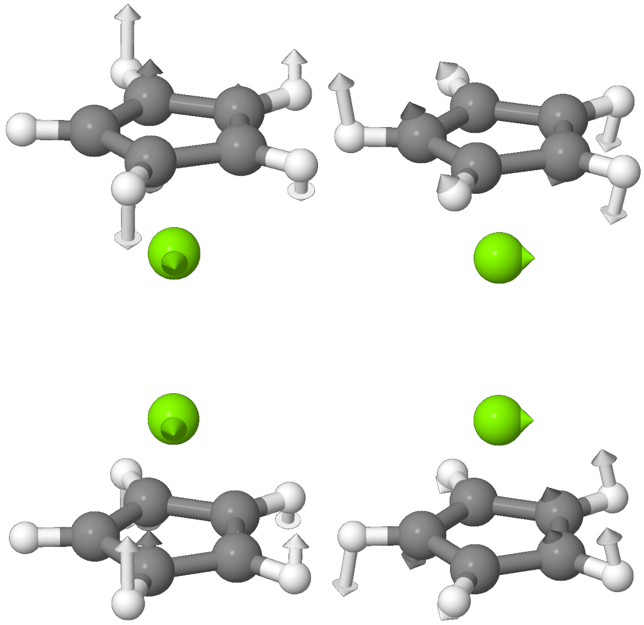

Figure 14: $\nu_{14}(e_1')$ modes at 254 cm^{-1}

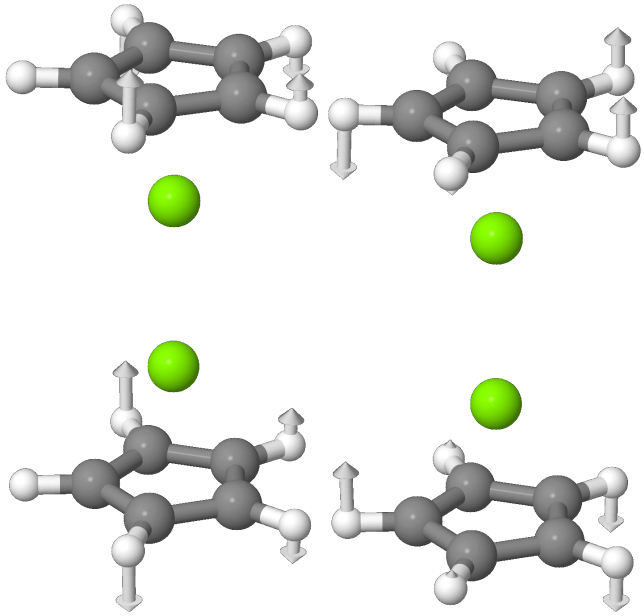

Figure 15: $\nu_{15}(e_1')$ modes at 778 cm^{-1}

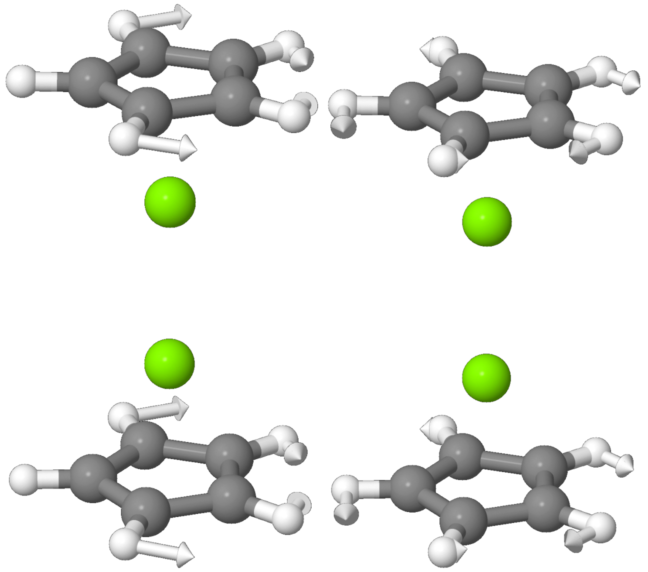

Figure 16: $\nu_{16}(e_1')$ modes at 1031 cm^{-1}

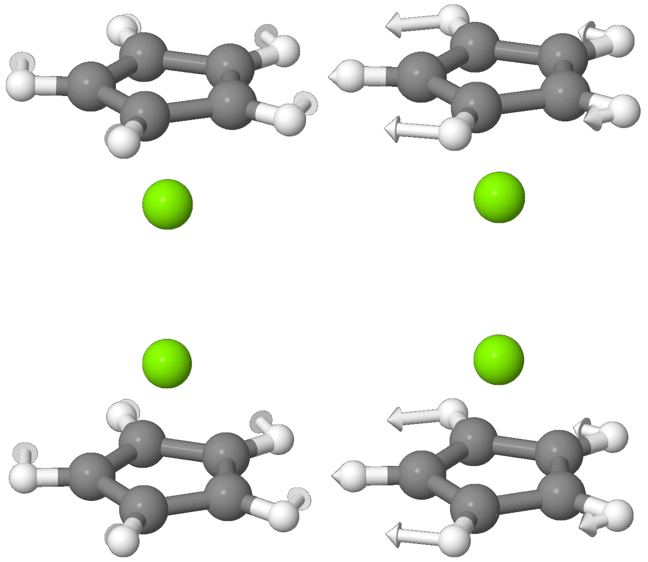

Figure 17: $\nu_{17}(e_1')$ modes at 1482 cm^{-1}

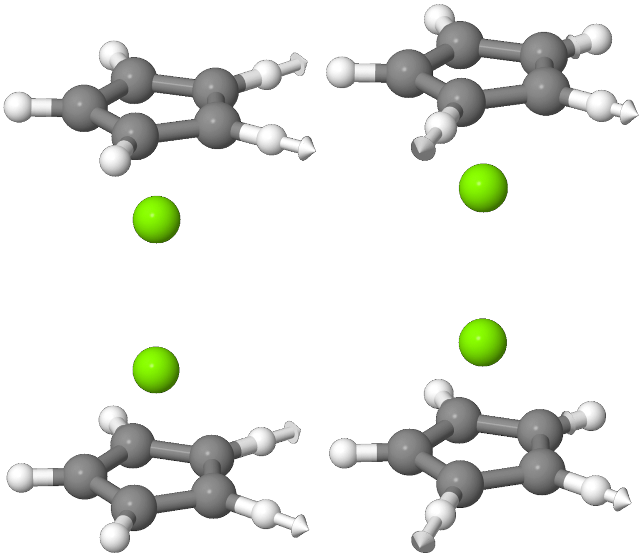

Figure 18: $\nu_{18}(e'_1)$ modes at 3264 cm^{-1}

Modes with E''_1 Symmetry

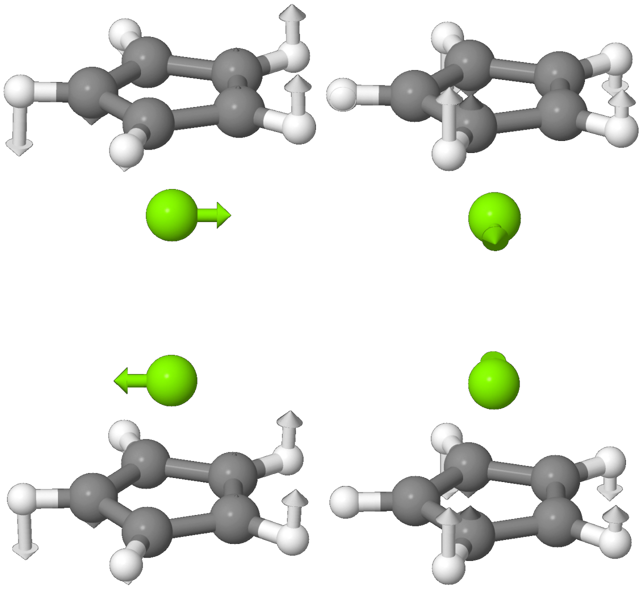

Figure 19: $\nu_{19}(e''_1)$ modes at 100 cm^{-1}

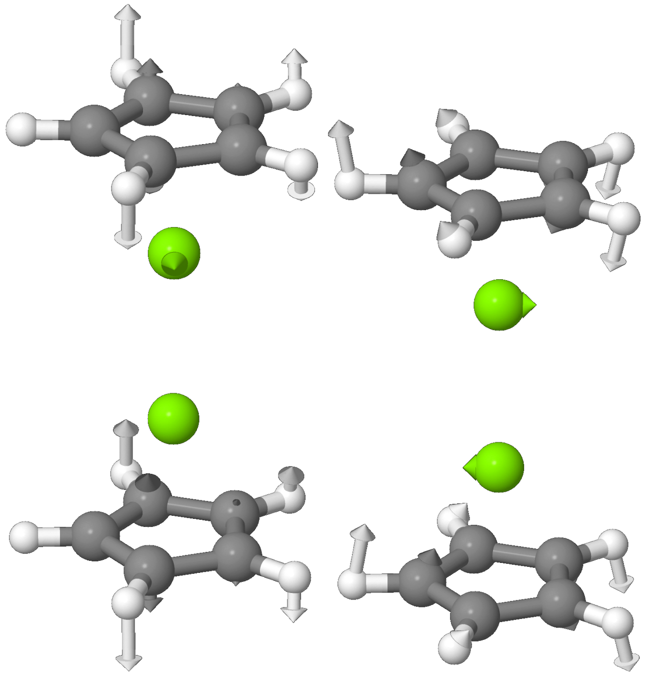

Figure 20: $\nu_{20}(e_1'')$ modes at 250 cm^{-1}

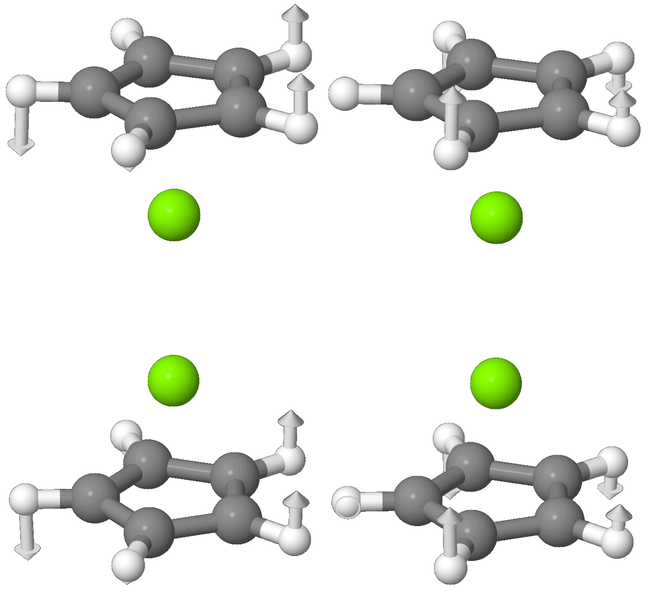

Figure 21: $\nu_{21}(e_1'')$ modes at 779 cm^{-1}

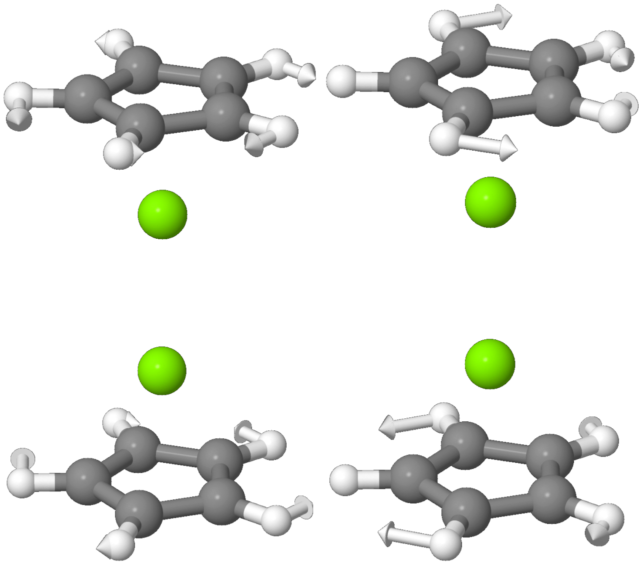

Figure 22: $\nu_{22}(e_1'')$ modes at 1031 cm^{-1}

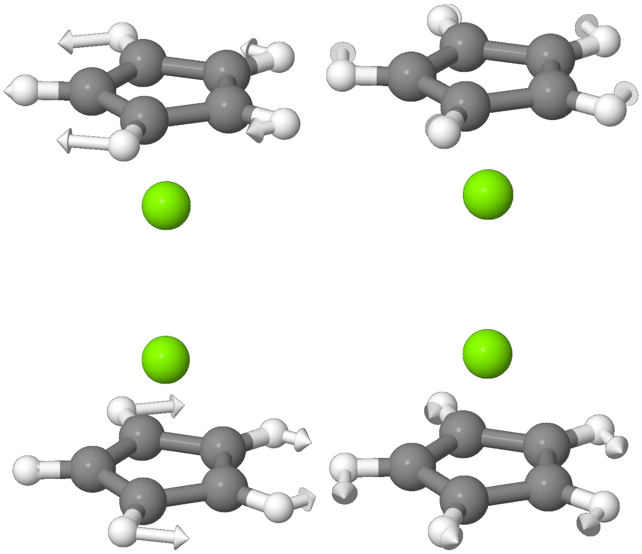

Figure 23: $\nu_{23}(e_1'')$ modes at 1483 cm^{-1}

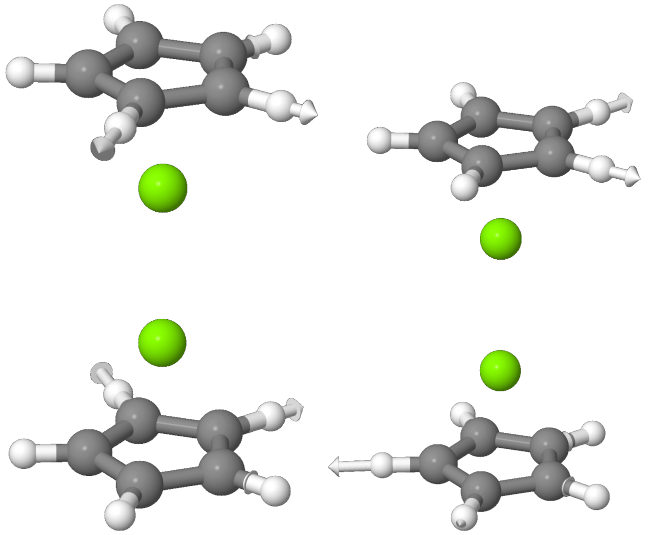

Figure 24: $\nu_{24}(e_1'')$ modes at 3264 cm^{-1}

Modes with E_2' Symmetry

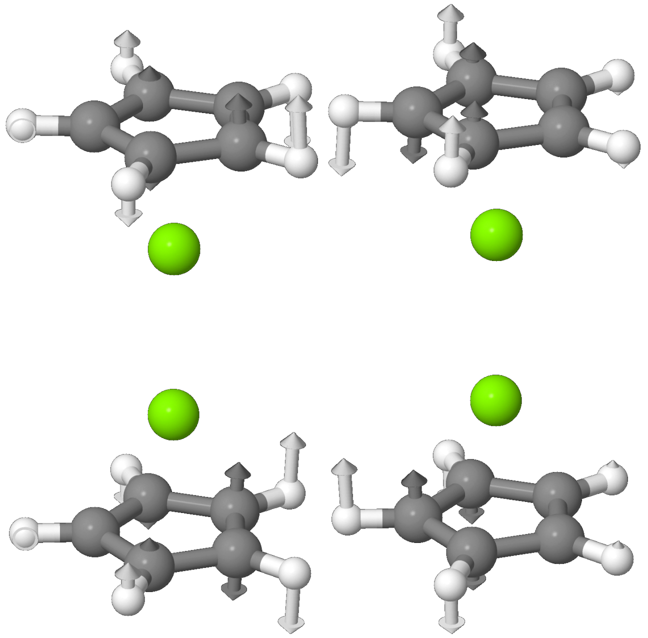

Figure 25: $\nu_{25}(e_2')$ modes at 626 cm^{-1}

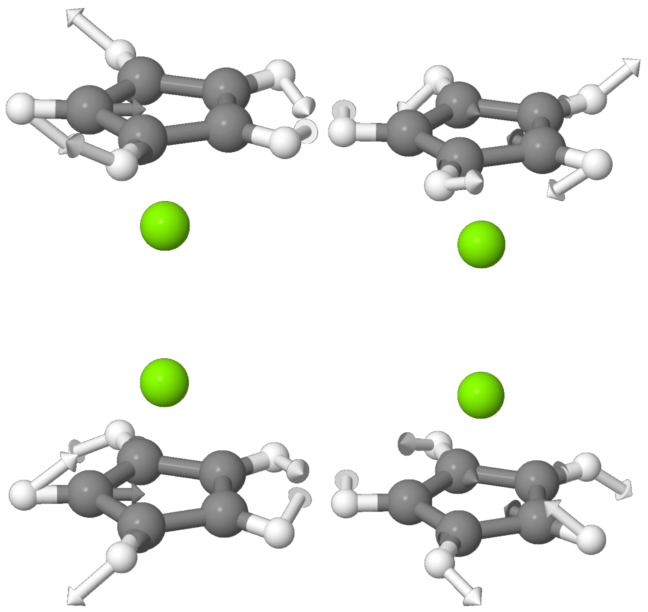

Figure 26: $\nu_{26}(e_2')$ modes at 860 cm^{-1}

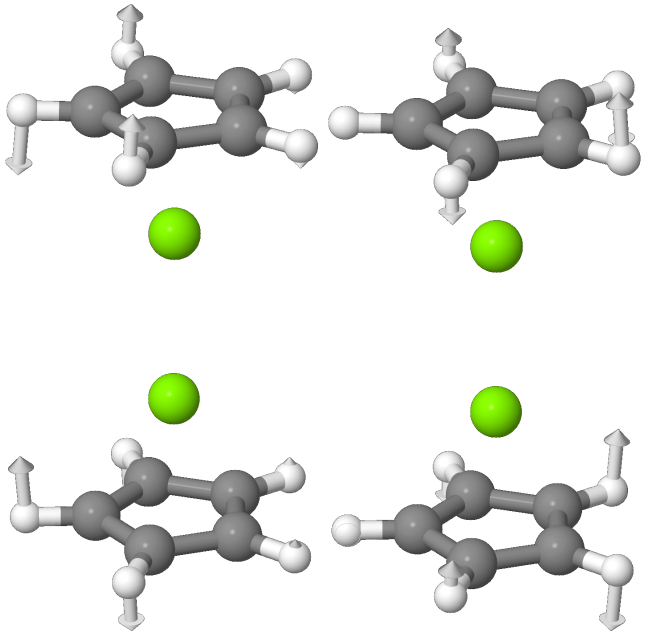

Figure 27: $\nu_{27}(e_2')$ modes at 885 cm^{-1}

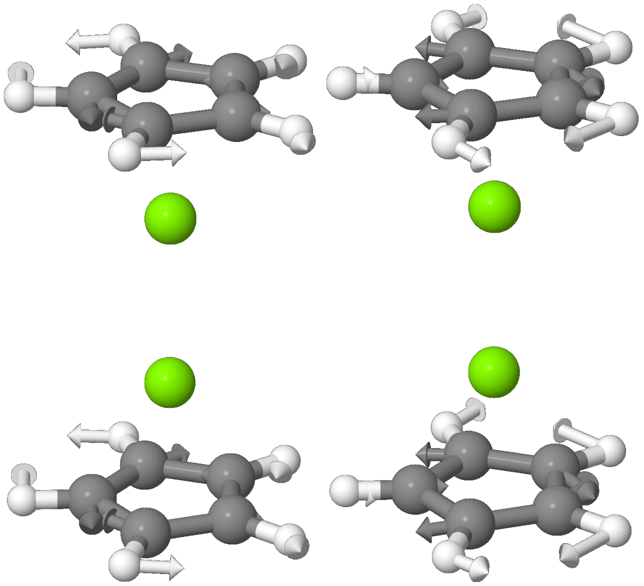

Figure 28: $\nu_{28}(e_2')$ modes at 1078 cm^{-1}

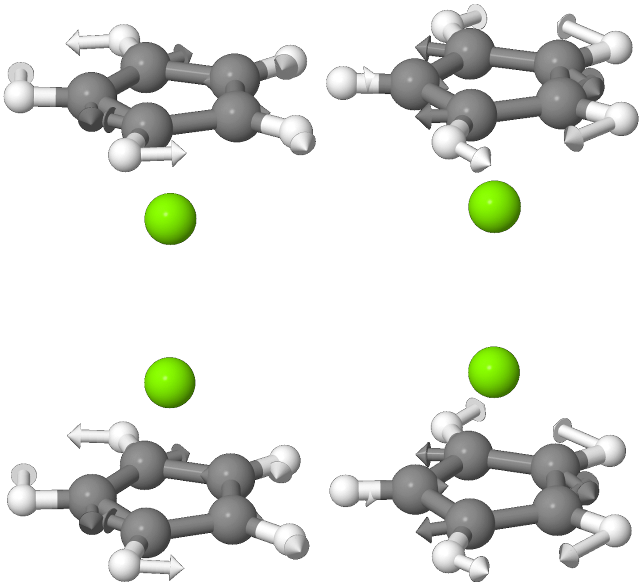

Figure 29: $\nu_{29}(e_2')$ modes at 1418 cm^{-1}

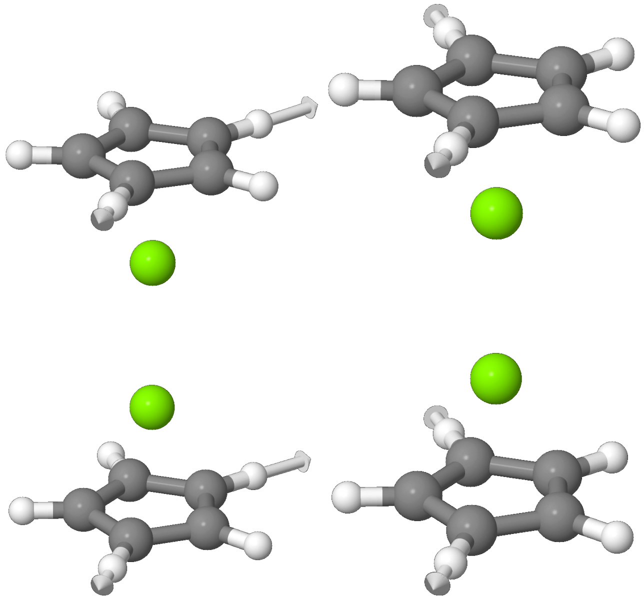

Figure 30: $\nu_{30}(e_2')$ modes at 3246 cm^{-1}

Modes with E_2'' Symmetry

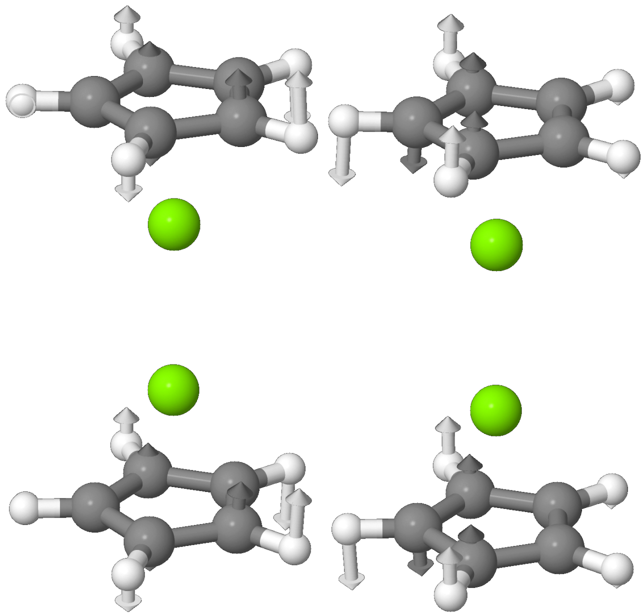

Figure 31: $\nu_{31}(e_2'')$ modes at 626 cm^{-1}

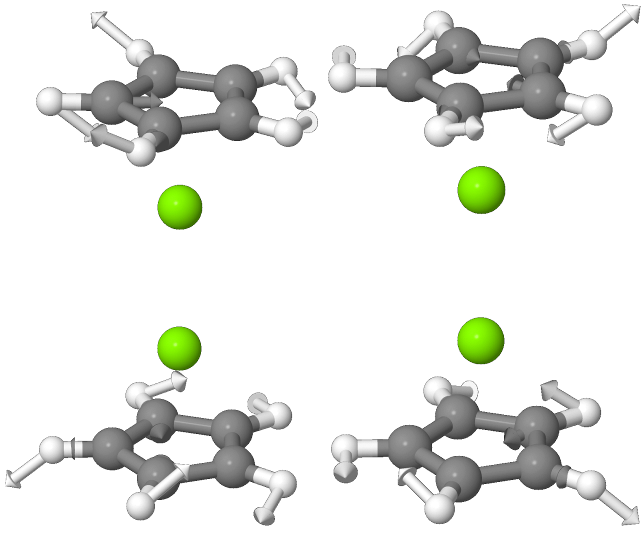

Figure 32: $\nu_{32}(e_2'')$ modes at 860 cm^{-1}

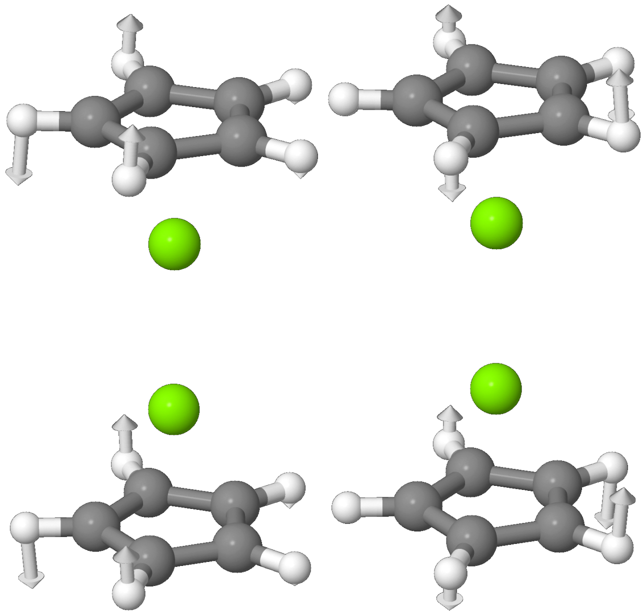

Figure 33: $\nu_{33}(e_2'')$ modes at 884 cm^{-1}

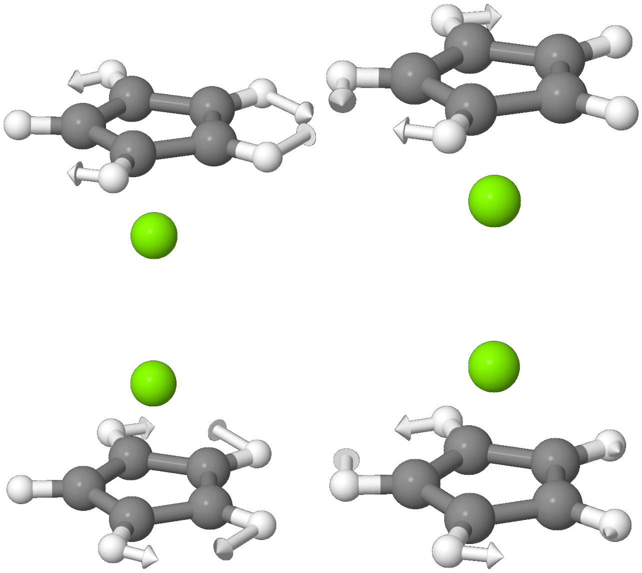

Figure 34: $\nu_{34}(e_2'')$ modes at 1078 cm^{-1}

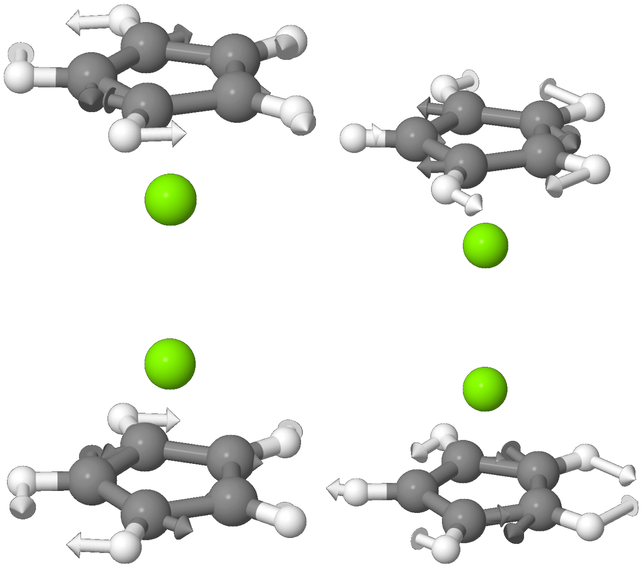

Figure 35: $\nu_{35}(e_2'')$ modes at 1418 cm^{-1}

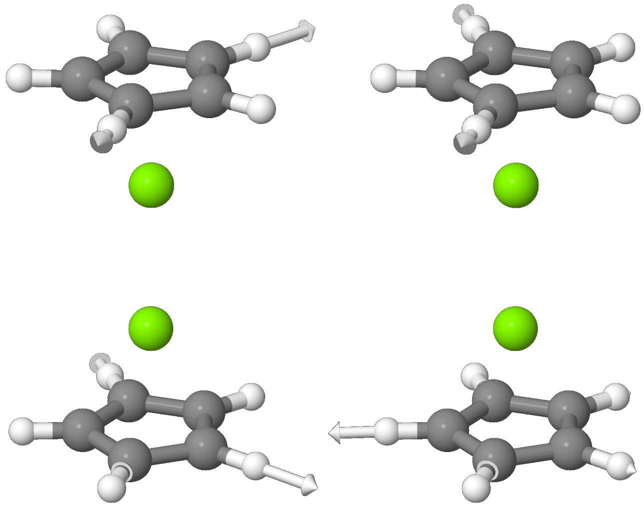

Figure 36: $\nu_{36}(e_2'')$ modes at 3245 cm^{-1}
